# Recent Advances in MOF-Based Materials for Biosensing Applications

**DOI:** 10.3390/s25082473

**Published:** 2025-04-14

**Authors:** Rudra Kumar, Muhammad Sajid Shafique, Sergio O. Martínez Chapa, Marc J. Madou

**Affiliations:** 1 School of Engineering and Sciences, Tecnologico de Monterrey, Av. Eugenio Garza Sada, NL, 2501, Sur, Monterrey 64849, Mexico; 2 Department of Mechanical and Aerospace Engineering, University of California Irvine, Irvine, CA 92697, USA

**Keywords:** metal–organic frameworks, MOF-derived carbon composites, MOF-derived metal oxides, sulfides, phosphides, MOF–noble metal composite, biosensing

## Abstract

Metal–organic frameworks (MOFs) or coordination polymers have gained enormous interest in recent years due to their extraordinary properties, including their high surface area, tunable pore size, and ability to form nanocomposites with various functional materials. MOF materials possess redox-active properties that are beneficial for electrochemical sensing applications. Furthermore, the tunable pore size and high surface area improve the adsorption or immobilization of enzymes, which can enhance the sensitivity and selectivity for specific analytes. Additionally, MOF-derived metal sulfides, phosphides, and nitrides demonstrate superior electrical conductivity and structural stability, ideal for electrochemical sensing. Moreover, the functionalization of MOFs further increases sensitivity by enhancing electrode–analyte interactions. The inclusion of carbon materials within MOFs enhances their electrical conductivity and reduces background current through optimized loading, preventing agglomeration and ensuring uniform distribution. Noble metals immobilized on MOFs offer improved stability and catalytic performance, providing larger surface areas and uniform nanoparticle dispersion. This review focuses on recent developments in MOF-based biosensors specifically for glucose, dopamine, H_2_O_2_, ascorbic acid, and uric acid sensing.

## 1. Introduction

Metal–organic frameworks (MOFs) are a class of materials that combine metal building units such as metal ions or metal clusters with organic ligands to form frameworks that can be used to tune their structures according to the requirements of various applications [1,2]. MOFs have outstanding properties, including a high surface area, highly ordered structure, crystalline nature, adjustable pore size, tunable structures, and high porosity. These unprecedented properties make MOFs a key material with ultrahigh porosity that can potentially supersede commercially available high surface area materials, such as zeolites and activated carbons [3,4,5,6]. Additionally, MOFs can be designed during the synthesis process to have altered physicochemical properties by varying the metal nodes, organic ligands, temperature, time, and other reaction conditions. These remarkable properties of MOFs have been widely used in numerous functional applications, such as gas storage, catalysis, energy storage devices, separation, chemical and biological sensors, heavy metal ion detection, and drug delivery, to name a few [5,6,7,8]. To further improve the physicochemical properties of MOFs, various composite materials have been synthesized with pristine MOFs and functional materials, such as carbon, metal nanoparticles, polymers, enzymes, and other conducting substrates, which can tune the mechanical, physical, chemical, optical, and electrical properties of pristine MOFs [9,10].

In recent years, various types of MOFs and their composites have been designed and developed with excellent catalytic performance; improved host and guest interactions; high stability in harsh conditions, such as low and high pH; and better signal transduction in chemical and biosensing, including microfluidic platforms and paper-based point-of-care devices [11,12,13,14]. A variety of MOF composites have been reported, such as MOF–carbon composites, MOF–nanoparticles, MOF–metal oxides, MOF–conducting polymers, and MOF–enzymes [15,16,17]. Furthermore, the developments of MOF derivatives show the advantages of structural optimization and component adjustment. This review is mainly focused on the recent developments and designs of MOF materials, such as novel MOF synthesis, MOF-derived carbon materials, and MOF nanocomposites such as MOF/metal oxide, MOF/metal sulfides, MOF/noble metals, and MOF/conducting polymers. We highlight the advantages of MOFs and their composites and further emphasize their potential applications in biosensing, especially in point-of-care devices, paper-based biosensing, flow-through biosensing, wearable sensors, biomedical devices, and in the detection of various types of ions, such as heavy metals and other pharmaceutical wastes, including various types of antibiotics. Lastly, we emphasize the future prospects of MOFs and their composites in biosensing applications.

## 2. MOFs and Their Derivatives

Hollow/porous carbon derived from MOFs:

Electrochemical sensing performance can vary depending on several factors, including the specific materials used, the fabrication methods, and the target analyte being detected. When comparing the performance of electrochemical sensors based on hollow/porous carbon derived from MOFs with other materials, several considerations come into play. Hollow/porous carbon derived from MOFs often exhibits unique properties such as a high surface area, tunable porosity, and excellent electrical conductivity. These characteristics can contribute to enhanced sensing performance by providing a large surface area for analyte interaction, the efficient mass transport of ions, and improved electron transfer kinetics. Additionally, the hierarchical pore structure of these materials can facilitate rapid analyte diffusion and increase the active sites available for sensing. Compared to other carbon-based materials commonly used in electrochemical sensing, such as graphene or carbon nanotubes, hollow/porous carbon derived from MOFs may offer advantages in terms of synthesis versatility, structural control, and scalability. However, the specific performance of an electrochemical sensor based on hollow/porous carbon derived from MOFs would depend on factors such as the target analyte, the sensor configuration, and the intended application. Overall, while hollow/porous carbon derived from MOFs holds promise for electrochemical sensing applications due to its unique properties, rigorous comparative studies are necessary to determine its performance relative to other materials in specific sensing scenarios.

Porous carbons with various architectures and morphologies derived from MOFs have been synthesized as electrode materials for electrochemical biosensors. The derived carbons possess a high surface area, hierarchical pores, excellent electrical conductivity, low density, and are non-toxic and environmentally friendly, making them suitable for use in electrochemical sensors that require good stability and target analyte adsorption capacity [18,19]. The general strategy for synthesizing porous carbon using MOFs involves high-temperature carbonization at 800–1000 °C in the absence of air, followed by etching to remove the embedded metal and form the final porous structures [18,20,21]. During the carbonization process, the organic part present in the MOF is converted into carbon and the porous structures are formed by the removal of the metal through the etching process. In this context, zinc-containing MOFs are commonly used as sacrificial templates to prepare porous carbon and nitrogen-doped porous carbon through high-temperature carbonization. The selection of different functional organic ligands during MOF synthesis is a major advantage in creating such high surface area porous carbons with heteroatom doping. Ligand selection plays a key role in tuning the porosity, pore size, and surface area of the derived porous carbon without the need for additional carbon or nitrogen sources. For example, mesoporous carbon materials derived from zeolitic imidazolate framework-8 (ZIF-8) were synthesized by high-temperature carbonization followed by acid leaching from an aqueous HCl solution to sense pentachlorophenol [22].

In another work, a ZIF MOF-derived nitrogen-doped porous carbon/multi-walled carbon nanotube composite (MWCNT/NPC) was reported for the detection of calcium ions. MWCNTs were attached to the ZIF in situ by mixing functionalized MWCNT, Polyvinylpyrrolidone (PVP), cobalt nitrate, and 2-methyl-imidazole (2-MeIM) in methanol at room temperature to form the ZIF-MWCNT nanocomposite, followed by carbonization and demineralization of inorganic impurities to construct the MWCNT/NPC composite. 2-methylimidazole was used as a nitrogen-containing organic ligand to provide nitrogen doping of the carbon nanocomposite. The resulting nanocomposite had a highly conductive MWCNT skeleton threaded with a mesoporous NPC framework to form a necklace-like structure that acted as a solid contact ion-selective electrode material with detection limit of 1.49 nM and a sensitivity of 25.87 ± 0.03 mV decade^−1^ for detecting calcium ions [23].

**Figure 1:**
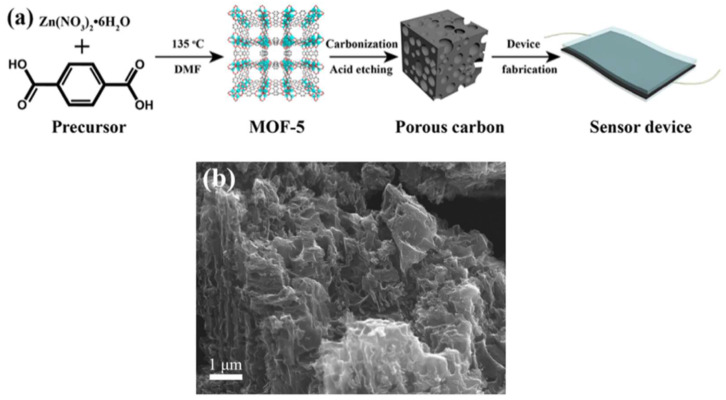
(**a**) Procedure for the synthesis of porous carbon from zinc-based MOF [18]. (**b**) SEM image of porous carbon derived from isoreticular metal–organic framework-8 (IRMOF-8) [25].

A zinc-based MOF precursor was used to synthesize a porous carbon and made a porous carbon–polydimethylsiloxane (PDMS) composite for use in a bimodal flexible sensor that could detect temperature and pressure simultaneously. The process of making a sensor device involved casting the PC/PDMS composite onto the ITO-coated PET substrate, which was then subjected to thermal treatment. Subsequently, another piece of ITO-coated PET substrate was placed on top, and copper conducting wires were affixed to the ITO electrodes (Figure 1a) [18]. The sensing operation relied on the change in contact area within the composite film and between the composite film and electrodes under external pressure, with the PC/PDMS composite film containing numerous meso- and micropores exhibiting increased resistance initially due to the limited contact area, which decreased as external pressure was applied, creating more conductive pathways through the compression of pores and through the enhanced contact between particles and electrodes, subsequently returning to initial resistance levels after pressure removal, thereby indicating that the pressure sensing capability of the PC/PDMS composite was predominantly influenced by variations in pore structure and contact area. Luo et al. synthesized nitrogen-doped porous carbon using a zinc-containing MOF with a high amount of nitrogen-based organic 2-methylimidazolate ligand [24].

Recently, Xiao et al. reported the synthesis of exfoliated porous carbon (EPC) by carbonizing IRMOF-8 at 1000 °C and then exfoliating the resulting porous carbon through ultrasonication (Figure 1b) [25]. The EPC material had a high surface area of 1854 m^2^ g^−1^ and showed good sensitivity towards chloramphenicol using square wave voltammetry. Lin et al. synthesized a metal–organic coordination polymer using two mixed organic bridging ligands, including bis-pyridyl-bis-amide (N,N′-di(3-pyridyl)adipoamide) ligand (3-dpaa) and 2,5-thiophene carboxylic acid (2,5-H_2_TDC) co-ligands using a hydrothermal method. This polymer was used as a catalyst precursor for growing carbon nanotubes using chemical vapor deposition, which was then used for detecting small molecules such as xylene and nitrobenzene [26].

## 3. Metal–Organic Framework Composites

Pristine MOFs have been modified to create various types of composites that combine the advantages of MOFs with the functional materials of the guest, such as gas molecules, metal ions, dyes, chromophores, biomolecules, etc. These MOF composites are innovative advanced materials that can overcome the limitations of pristine MOFs. Nowadays, several types of MOF composite materials have been synthesized, such as MOF/metal oxides, MOF/conducting polymers, MOF/noble metals, and MOF/metal sulfides, among others. These nanocomposites have been extensively used in various electrochemical applications, including sensing biomolecules, detecting heavy metal ions, and other advanced applications. This section presents the synthesis of metal MOF composites and their advantages in advanced biosensors.

### 3.1. MOF/Carbon Composite

Pristine MOFs typically suffer from poor conductivity, low stability, and limited redox activity, which hinder their direct use as electrode materials in electrochemical sensors. Coupling MOFs with highly conductive materials is an effective strategy to overcome these limitations and enhance their electrochemical performance. Carbon, being a highly conductive material, can be mixed with MOFs to increase its electrical conductivity, to reduce background current by a controlled and uniform distribution of carbon within the MOF matrix to prevent the agglomeration or clustering of carbon particles, or by optimizing carbon loading to minimize the overcrowding of conducting pathways or inadequate conductivity enhancement, to provide electroactive sites for target molecule recognition, and improving sensitivity and selectivity combined with a fast response time. For example, carbon paste electrode (CPE)-modified MOF composites provide a wide electrochemical potential window ranging from −1.4 to +1.3 V vs. SCE and offer simple preparation and the easy regeneration of the surface. This makes them an alternative to the hanging drop mercury electrode for the detection of various types of target analytes through electrochemical methods [27].

**Figure 2:**
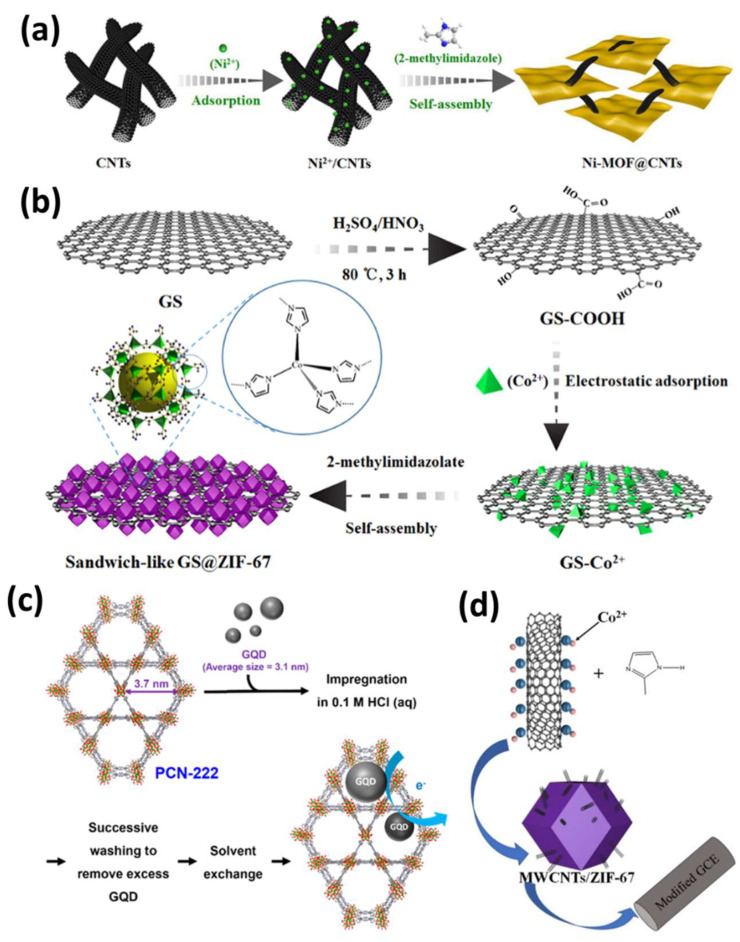
(**a**) Synthesis process of the Ni-MOF@CNTs heterogeneous composites [29]. (**b**) Fabrication of sandwich-like GS@ZIF-67 hybrids [31]. (**c**) Incorporation of GQD into PCN-222 via direct impregnation [32]. (**d**) Preparation of MWCNTs/ZIF-67-modified electrode [36].

In this context, various types of MOFs such as Cu-MOF [28], Ni-MOF [29], Fe-MOF [30], Co-MOF [31], Zr-MOF [32], Mn-MOF [33], etc., have been modified with carbon materials that have shown improved sensing towards multiple analytes/biomolecules (Figure 2a–c) [34]. Xu et al. prepared a 3D interlinked network of the Ni-MOF@CNTs hybrid material using quasi 2D Ni-MOF nanosheets and CNTs through in situ synthesis. The resulting material exhibited excellent electrochemical sensing performance for toxic bisphenol in various real samples, with a concentration range of 1 nM to 1000 nM with a detection limit of 0.35 nM. The large surface area of 2D Ni-MOF provided multiple active sites, while CNTs prevented the restacking of 2D NI-MOF and possessed excellent electrical properties for outstanding electrochemical performance (Figure 2a) [29]. Further, Li et al. immobilized ZIF-65 MOF-containing redox-active nitro groups on carboxylated CNTs for the electrochemical sensing of ascorbic acid, where nitro groups in the organic ligand acted as a sensing unit and CNTs enhanced the electrical conductivity with the detection limit of 1.03 mM [35]. Zheng et al. reported a porous ZIF-67/MWCNTs nanocomposite for hydroquinone and catechol detection using the DPV method in the linear range between 0.5 and 100 μM, as shown in Figure 2d [36].

Apart from forming nanocomposites of MOFs with CNTs, graphene nanosheets have also been utilized as conducting electrode materials for synthesizing MOF-based nanocomposites [37,38]. In this regard, Chen et al. synthesized a sandwich-like GS@ZIF-67 hybrid through the in situ loading of ZIF-67 onto graphene nanosheets, enhancing their electrochemical activity for glucose sensing. The sensor exhibited a sensitivity of 1521.1 μA mM^−1^ cm^−2^, a detection limit of 0.36 μM, and excellent stability, successfully detecting glucose in human serum (Figure 2b) [31]. Additionally, Chen et al. impregnated graphene quantum dots onto porphyrinic zirconium Zr-MOF, facilitating donor–acceptor charge transfer from the graphene quantum dots to the porphyrinic linkers for nitrite sensing (Figure 2c) [32].

### 3.2. MOF-Derived Carbon Noble Metal Composites

Noble metal-decorated MOF-derived carbon nanocomposites are promising materials in electrochemical and optical sensors due to their improved electrical conductivity, high surface area, unique material structure, and good catalytic activity. There are three basic synthesis routes for noble metal nanoparticles (NPs) on MOF-derived carbon: immobilizing noble metals on the outer surface of MOFs, depositing them into the pores of MOFs, and enclosing them inside the porous MOFs, followed by the carbonization process at a high temperature in an inert atmosphere. The resulting MOF-derived carbon–noble metal nanocomposites have several advantages, such as protecting against agglomeration, confining growth, improving stability, enhancing conductivity, and uniformly dispersing NPs, which provide a larger surface area and better catalytic and sensing abilities for the composite [39,40,41,42].

Moreover, noble metals can exhibit enzyme-like behavior, known as nanozyme activity, and are utilized for the detection of various analytes. Noble metal nanoparticles can mimic natural enzymes, including peroxidases, oxidases, and catalases. In this context, Chen et al. used a Pt NP-modified Fe-MIL-88 MOF composite as a peroxidase mimic and demonstrated a dual readout system with both electrochemical and colorimetric signals for thrombin detection. Additionally, a gold NP initiator was added to enhance the dual signals and trigger the hybridization chain reaction between the thrombin aptamer and its complementary sequence immobilized on the gold electrode, enabling the detection of thrombin [43]. Additionally, The UIO-66-NH_2_ MOF-Pt NP composite was reported to exhibit peroxidase-like activity, facilitating the oxidation of 3,3′,5,5′-tetramethylbenzidine in the presence of hydrazine. However, upon interaction with Hg^2+^ ions, the composite effectively suppressed its peroxidase-like activity, enabling the detection of Hg^2+^ with a detection limit of 0.35 nM [44].

Recently, Aijaz et al. used a double-solvent approach to immobilize ultrafine Pt NPs into the hydrophilic pores of the Cr-based MIL-101 MOF. The double solvent method was employed to prevent nanoparticle aggregation, utilizing a hydrophilic solvent with metal precursor volume equal to or less than MIL-101’s pore volume, while a larger quantity of hydrophobic solvent aided in suspension. Capillary force facilitated the penetration of aqueous H_2_PtCl_6_ into the hydrophilic pores, minimizing deposition on the outer surface, unlike in conventional methods [45]. This approach resulted in uniformly distributed Pt NPs inside the pores of the MOF, preventing agglomeration on the outer surface of the MOF. This method was advantageous over the conventional single solvent impregnation process that deposited and agglomerated metal NPs on the outer surface of the MOF in the drying step.

**Figure 3:**
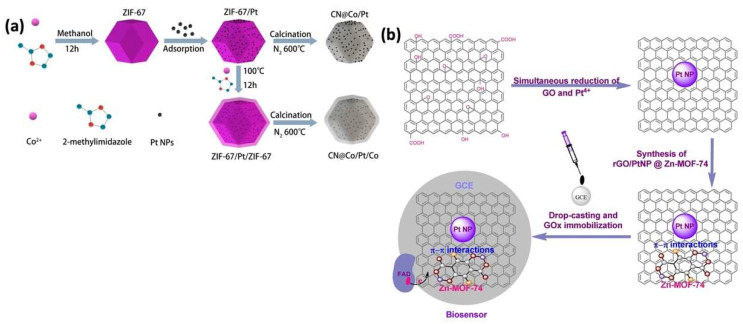
(**a**) Description of the typical synthesis approach of ZIF-67/Pt, ZIF-67/Pt, CN@Co/Pt, and CN@Co/Pt/Co nanocomposites [46]. (**b**) Representation of the assembly process of the modified GCE with GOx-rGO/Pt NPs@Zn-MOF-74 [47].

Xu et al. utilized ZIF-67 MOF, with cobalt as the metal precursor and 2-methylimidazole as an organic ligand, to encapsulate homogeneously distributed Pt NPs. The structure was then covered with a ZIF-67 shell to form a core–shell architecture, followed by a calcination process in a nitrogen atmosphere to create a novel hybrid nanocomposite as a nitrogen-doped carbon on cobalt/Pt/cobalt (CN@Co/Pt/Co) for the electrochemical detection of dopamine. After pyrolysis, the ZIF-67 was converted into N-doped porous graphitic carbon embedded with cobalt nanoparticles, which enhanced electron conductivity, prevented Pt NP agglomeration, and improved the surface area by reducing particle size and improving NP dispersion during calcination. The electrochemical sensing performance of the CN@ Co/Pt/Co nanocomposite electrodes, evaluated using differential pulse voltammetry (DPV), showed a good sensitivity of 17.38 μA μM^−1^ cm^−2^ and a detection limit of 2.75 nM for dopamine (Figure 3a) [46].

A similar study was conducted by Li et al., who investigated the development of a new hybrid nanocomposite, CN@Co/Pt/Co, for the highly sensitive electrochemical biosensing of dopamine. In this process, Pt NPs were first evenly distributed on the surface of ZIF-67’s surface, followed by the formation of a core–shell structure by encapsulating them within a ZIF-67 shell. Finally, heating the ZIF-67/Pt/ZIF-67 composites under N_2_ at 600 °C for 2 h resulted in the formation of CN@Co/Pt/Co nanocomposites.

Uzak et al. synthesized a Pt NP/rGO-MOF hybrid nanocomposite material, which was used as a glucose biosensor. The composite came with improved conductivity, high porosity, and an enlarged surface area, which facilitated the immobilization of glucose oxidase and resulted in a detection limit of 1.8 μM and a sensitivity of 64.51 μA mM^−1^ cm^−2^ during glucose sensing (Figure 3b) [47].

Apart from platinum, silver nanoparticles (Ag NPs) were also utilized in the voids of MOF to enhance electrical conductivity, surface area, and sensing performance. In this context, Bin et al. developed Ag NPs decorated with ZIF-8 MOF for the electrochemical sensing of chloride ions with a detection range of 5 to 4000 μmol and a detection limit of 0.61 μmol [39].

In addition to silver, gold NPs were reported to have electrocatalytic performance towards various sensors and were confined in porous MOFs to enhance electrical conductivity, selectivity, and sensitivity [48,49]. Zhang et al. used a ZIF-67 MOF as a template for the effective confinement of Au NPs and as a source for nitrogen-doped porous carbon to construct a ternary hybrid structure comprising gold NPs embedded in N-doped porous carbon attached to rGO nanosheets using a wet chemical method followed by pyrolysis. This ternary hybrid structure exhibited the enhanced detection of hydrazine in both liquid and gas phases due to the synergistic effect of all three components, including improved electrocatalytic activity from the gold NPs, the facilitation of electron transfer from two-dimensional rGO nanosheets, and the increased surface area from the MOF-derived nanoporous carbon [50].

### 3.3. MOF-Derived Metal Oxide Composites

MOF-derived metal oxides:

MOF-derived metal oxides can be produced through calcination at high temperatures in the presence of air, without damaging the morphology and porous structure of the parent MOF. Recently, Arul et al. fabricated CuO nanorods on a GCE through an electrochemical deposition method using Cu_3_(BTC)_2_ (BTC: benzene tricarboxylate) MOF as a precursor. These nanorods were then utilized for the electrochemical oxidation of glucose [51].

**Figure 4:**
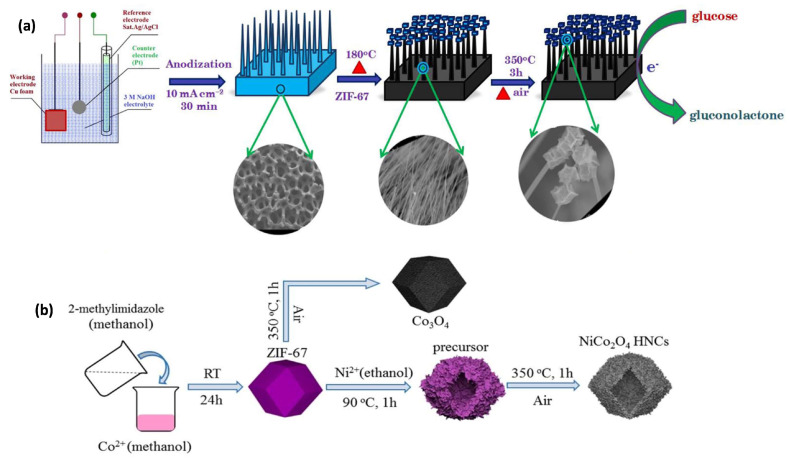
(**a**) Illustration of the 3D CuO NWs@Co_3_O_4_ fabrication process and its role in glucose oxidation catalysis [52]. (**b**) Representation of the synthesis process for NiCo_2_O_4_ hollow nanocages (HNCs) and Co_3_O_4_ [57].

Apart from monometallic oxides, mixed metal oxides and ternary metal oxides exhibit higher electrical conductivity, better electrochemical and optical sensing ability, fast responses, high sensitivity, and low detection limits due to the synergistic effect of more than one metal component and their complex chemical composition. Muthurasu et al. fabricated copper oxide nanowires in a cobalt oxide (CuO NWs@Co_3_O_4_) composite, which was used as a binder-free electrode for nonenzymatic glucose sensors. The CuO NWs were grown on a conducting electrode using electrochemical anodization techniques, and cobalt-based ZIF MOF was deposited on the surface of the CuO NWs through a solution deposition method. The ZIF was converted to Co_3_O_4_ after a heat treatment process, as shown in Figure 4a [52].

One study utilized CuOx@Co_3_O_4_ core–shell nanowires on a conducting Cu foam substrate, where the ZIF-67 MOF was attached to Cu(OH)_2_ wires and then calcined at high temperatures to form CuOx nanowire cores and Co_3_O_4_ nanoparticle shells [53]. This core–shell composite electrode demonstrated a synergistic catalytic effect, enhancing the electrocatalytic oxidation and detection of glucose in human blood serum. The resulting glucose sensor demonstrated a linear range, sensitivity, detection limit, and response time of 0.1–1300.0 μM, 27,778 μA mM^−1^ cm^−2^, 36 nM, and approximately 1 s, respectively. Additionally, various metal oxides, such as Fe_2_O_3_, NiO, and ZnO, can be deposited on CuOx NWs using their respective MOF precursors. Spinel cobaltites, including NiCo_2_O_4_, CuCo_2_O_4_, ZnCo_2_O_4_, and MnCo_2_O_4_, have also been utilized in electrochemical sensors and biomolecule detection due to their multivalent electronic states, enhanced electrical conductivity, and the synergistic effects of multiple metal species [54,55,56].

The general synthesis procedure for hollow/porous mixed metal oxide materials involves two steps: first, one of the metals is used to form an MOF in the desired architecture, while the second metal is deposited on the surface or inside of the first metal-derived MOF through hydrothermal or solvothermal methods. The resulting mixed metal precursor is then converted to the desired crystalline phase using a high-temperature calcination process. These MOF derivatives improve the catalytic and sensing performances by alleviating the poor conductivity and structural instability of the original MOFs. For instance, Cui et al. synthesized hollow mesoporous CuCo_2_O_4_ microspheres using a hydrothermal method followed by high-temperature carbonization, where copper- and cobalt-based MOFs were used as templates. The resulting MOF derivatives exhibited excellent catalytic performance towards H_2_O_2_, with a sensitivity of 654.23 μA mM^−1^ cm^−2^ and a detection limit of 3 nM [56]. Similarly, Feng et al. developed NiCo_2_O_4_ hollow nanocages via a solvothermal method followed by a calcination process, using ZIF-67 MOF and a nickel precursor, and used them as promising catalysts for glucose oxidation (Figure 4b) [57]. Zhang et al. used a Zn-Co MOF precursor to synthesize a porous ZnCo_2_O_4_ micro-rice-like structure deposited on a glassy carbon electrode for non-enzymatic glucose sensing, demonstrating improved electrocatalytic properties and higher electrical conductivity compared to Co_3_O_4_-based electrodes [58].

Layered double hydroxide (LDH) is another promising candidate for biosensors due to its biocompatibility, non-toxicity, and having more active sites than single metal hydroxides for catalysis. Recently, Song et al. developed a binder-free electrode on a conducting carbon cloth decorated with cobalt carbonate nanorod@MOF-derived NiCo LDH (CC@CCH MOF LDH) nanosheets for non-enzymatic glucose detection. The CC@CCH three-dimensional structure was synthesized via a hydrothermal method, with the growth of cobalt carbon nanorods on carbon cloth followed by the synthesis of CC@CCH MOF using 2-methyl imidazole ligand. The cobalt source and sacrificial template were used to synthesize CC@CCH MOF LDH in the presence of a nickel precursor [59].

### 3.4. MOF/Conducting Polymer Composites

Conducting polymers possess outstanding electrical properties and are widely used in various sensing technologies owing to their ability to exhibit redox behavior at specific electrochemical potentials [60]. When conducting polymers are merged with non-conducting MOFs, the resulting composite not only shows promising electrical, mechanical, optical, electrocatalytic, and sensing performance by utilizing the advantages of conducting polymers, such as their biocompatibility, low cost, high conductivity, good stability, and ease of synthesis, but also mitigates the drawbacks of pristine MOFs, including their non-conducting behavior and structural fragility [61,62,63].

**Figure 5:**
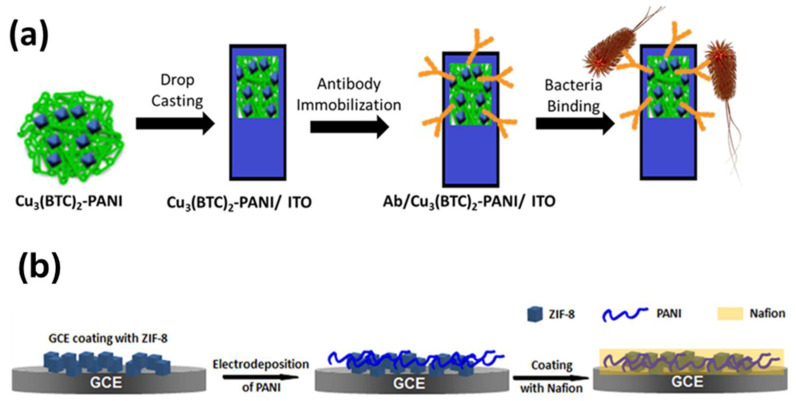
(**a**) Schematic showing the bioelectrode fabrication process and its application in the impedimetric detection of *E. coli* [64]. (**b**) Fabrication process of Nafion/PANI/ZIF-8 nanocomposites [67].

Moreover, by taking advantage of the benefits of MOFs, such as their high surface area and increased adsorption sites, the resulting nanocomposite serves as a backbone for conducting polymers, which speeds up electron/ion diffusion in the matrix. Generally, conducting polymers are synthesized in situ through the chemical oxidative polymerization of respective monomers in the presence of MOFs. Recently, Cu-based MOFs were mixed with polyaniline (PANI) to enhance the electrical conductivity of the Cu_3_(BTC)_2_/PANI composite and to form a homogenous thin film on the electrode surface for the sensitive detection of *E. coli* (Figure 5a) [64]. Wang et al. prepared UiO-66-NH_2_/PANI by the polymerization of PANI on the surface of UiO-66-NH_2_ MOF, which was derived from hexameric Zr_6_O_32_ units and the 2-amino-terephthalate ligand for the electrochemical detection of Cd^2+^ ions in the concentration range of 0.5–600 mg L^−1^ with a limit of detection of 0.3 mg L^−1^. The metal cations were chelated with the amine groups to realize the electrochemical detection of Cd^2+^ ions [65].

Hou et al. synthesized a core−shell-structure of the MIL-101@cPANI composite where chiral PANI was in situ coated on the Cr-based MIL-101 MOF to integrate the conductivity and porosity with chirality for the chiral recognition and sensing of enantiomers such as L-carvone and D-carvone. The developed composite had the advantages of conducting chiral PANI in sensing performance, and having a high surface area MOF for better adsorption and enantioselectivity towards carvone enantiomers than that of chiral PANI alone [66]. Yuan et al. developed a Nafion/PANI/ZIF-8 composite through the electrochemical deposition and polymerization of aniline on the surface of ZIF-8-coated GCE followed by Nafion coating for the detection of dopamine in PBS, as shown in Figure 5b [67]. Nafion is a cation exchange polymer that acts as an electrode protection and improves ionic conductivity and that, combined with the high surface area MOF and PANI, imparts a synergistic effect towards the electrocatalytic performance of DA and better interference properties towards ascorbic acid. The above discussion suggests that MOF-conducting polymer composites have the potential to be used in various applications such as the fabrication of fascinating structures with improved properties like sensitivity, and in LOD, biochemical sensors, heavy metal ion detection, clinical diagnostics, and point-of-care devices.

### 3.5. MOF/Metal Sulfides/Phosphides, and Nitrides Composites

MOF-derived metal sulfides, phosphides, and nitrides have higher electrical conductivity compared to their oxides. Additionally, MOFs can act as bifunctional materials, serving as both a metal precursor and a sacrificial template to form hollow/porous nanostructures with nanovoids and functional shell metal sulfides, oxides, or nitrides without compromising the structure’s stability. In this section, we will review the recent progress and applications of these materials in biosensors.

#### 3.5.1. MOF-Derived Metal Nitrides

Transition metal nitrides (TMNs) are known for their high electrical conductivity, high melting point, thermal stability, and corrosion resistance [68]. Additionally, these materials possess outstanding electrochemical, catalytic, and optoelectronic properties [69,70,71].

**Figure 6:**
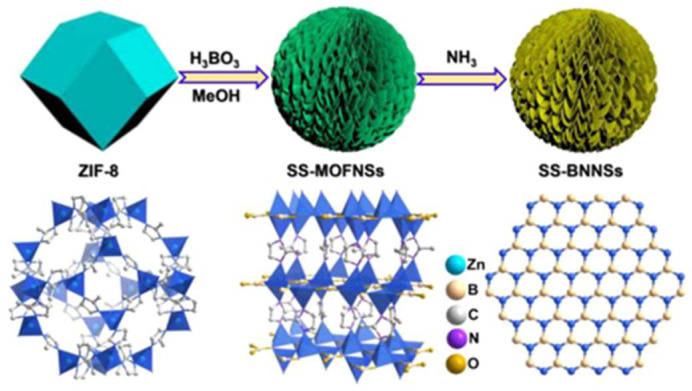
Schematic representation of the synthesis of SS-MOFNSs and SS-BNNSs [73].

However, synthesizing transition metal nitrides at low temperatures and pressures can be challenging due to the unreactive nature of most nitrogen-containing sources, including nitrogen and ammonia. At a high temperature and pressure, nitrogen atoms diffuse into the metal lattices of transition metals, combining the properties of covalent solids and ionic crystals [72]. MOF-derived TMNs possess hierarchical structures with high porosity, an outstanding surface area, and excellent electrical conductivity. Recently, Cao et al. synthesized a spherical-shaped boron-containing metal nitride constructed with sheet-like morphological units using MOF as a sacrificial template (Figure 6) [73]. The fabricated 3D hexagonal boron nitride structure had more active sites and a higher surface area necessary for catalytic processes. Apart from pure metal nitrides, composites with carbon have also been synthesized [71]. In this context, Chen et al. developed a nickel nitride carbon composite (Ni_3_N@C) through the direct pyrolysis of Ni-BTC MOF as a precursor for electrocatalytic glucose oxidation [74]. The resulting composite exhibited intrinsic electronic properties, primarily due to its fast charge transfer ability and higher electrical conductivity compared to metal oxides. The synergistic effect enhanced its electrochemical sensing performance, leading to a low detection limit, high sensitivity, and a wide linear detection range, which could be attributed to the metallic Ni_3_N structure and the nitrogen-containing organic linker.

#### 3.5.2. MOF-Derived Metal Sulfides

Transition metal sulfides (TMSs) are 2D layered structures in which metal atoms from groups 3 to 12 are sandwiched between two sulfur atoms, either through weak van der Waals forces or strong chemical bonding [75,76]. These materials are promising for biosensor applications due to their appropriate bandgap and large active sites that can bind to target bio-analytes or specific biomolecules. Moreover, TMSs exhibit better electrical conductivity compared to their oxide counterparts, enhancing charge transport for fast recognition and improved electrochemical sensitivity. The suitable bandgap also bestows better optoelectronic, photoluminescence, and electroluminescence properties for biosensing [77,78].

**Figure 7:**
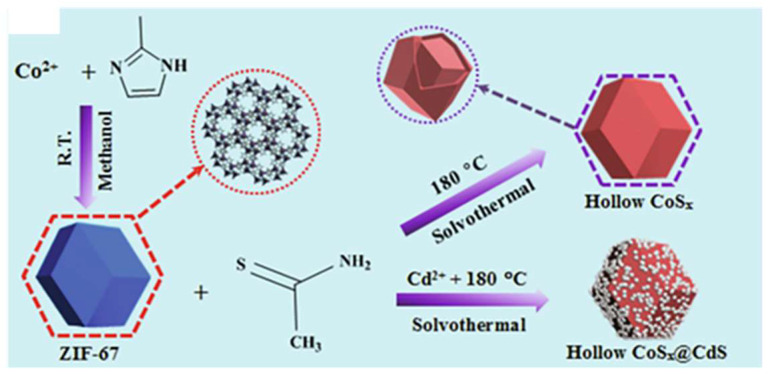
ZIF-67-derived CoS_x_ polyhedrons and CoS_x_@CdS composites [79].

In this regard, Zhang et al. used ZIF-67 polyhedra as a template and cobalt as a precursor to fabricate the CoS_x_@CdS heterojunction photocatalyst through the hydrothermal method, where CdS nanoparticles were grown on the cobalt sulfide surface (Figure 7) [79]. The CoS_x_@CdS-coated ITO electrode showed an enhanced photocurrent response towards Hg^2+^ ions compared to individual CoS_x_ and CdS electrodes, owing to the synergistic effects of cadmium sulfide decorated on cobalt sulfide, the large number of active sites that enhanced photoabsorption and redox reactions due to the hollow structure of CoS_x_, as well as the selective ion exchange and suppression of photogenerated electron-hole pair recombination. The photoelectrochemical sensor demonstrated the ability to detect the Hg^2+^ ion concentration in a wide linear range from 0.010 to 1000 nM [79].

Apart from the synthesis of single and heterostructure metal sulfides from MOF, multi-metallic MOF and LDH-derived metal sulfides have been reported recently, which can enhance electrical conductivity, reduce the bandgap, and provide abundant electrochemical active sites [80,81,82]. Additionally, layered hydroxides can boost ion intercalation, decrease mass transport resistance, and improve the sensitivity and response time of target molecule detection. Table 1 summarizes a comparative analysis of MOF-derived oxides and sulfides, presenting various materials along with their sensitivity, detection limits, and other key parameters listed.

#### 3.5.3. MOF-Derived Metal Phosphides

**Figure 8:**
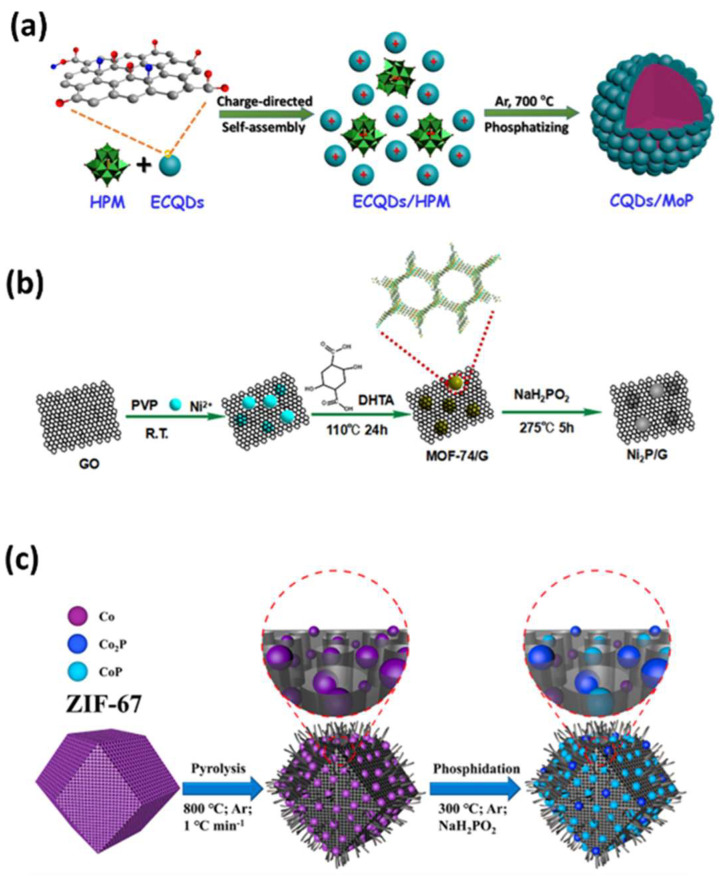
Phosphorization of MOF into (**a**) CQDs/MoP nanohybrid [91]. (**b**) Synthesis of Ni_2_P/graphene [92]. (**c**) Synthesis of CoP_x_@NCNTs’ polyhedrons [97].

Transition metal phosphides (TMPs) are materials with metal phosphide bonds that dissociate molecules through weak ligand effects, providing abundant catalytic active sites to bind target molecules. They have been reported as efficient electrocatalysts, electrochemical biosensors, and heavy metal ion detectors, as shown in Figure 8a [88,89,90,91,92,93]. The high electrocatalytic activity and electrochemical sensing performance of metal phosphides mainly depend on their hierarchical nanostructures, which facilitate better mass diffusion, and their electrically conducting frameworks, which improve electron transport [94]. The sensing performance of metal phosphides can be further enhanced by amalgamation with highly conducting porous carbon materials and heteroatom doping.

ZIF MOFs are three-dimensional porous coordination polymers comprising organic ligands with a high carbon, nitrogen, and metal content that can be used as precursor materials for the synthesis of hierarchical porous and functional phosphide derivatives [88]. In this framework, Xiao et al. utilized an amorphous Zn(PO_4_)_x_ sheet-like structure as a sacrificial template and cobalt-based ZIF-67 MOF as a carbon source to first synthesize spherical Zn(PO_4_)_x_@ZIF-67 composites and then convert them into nitrogen-doped porous carbon microspheres (Co_x_P/NC) through simultaneous carbonization and phosphorization in a single step [93].

Cobalt phosphide has been widely reported as an electrode material due to its high electrical conductivity, catalytic activity, and better durability [95,96]. The developed Co_x_P was a mixture of CoP and Co_2_P nanoparticles that were embedded in a three-dimensional nitrogen-doped graphitic carbon microsphere with a large surface area of 826 m^2^ g^−1^, uniform doping, good conductivity, and abundant pores. The GCE-loaded Co_x_P/NC composite exhibited good electrochemical detection towards 4-nitrophenol (4-NP) with a low detection limit of 2 × 10^−9^ mol L^−1^ and a high sensitivity of 20.9 μA μmol^−1^ L. Wang et al. prepared a nitrogen-doped porous carbon polyhedron embedded with cobalt phosphide nanoparticles and entangled with carbon nanotubes (CoP_x_@NCNTs) using pyrolysis in argon at a high temperature and phosphidation in the presence of NaH_2_PO_2_ from ZIF-67 (Figure 8c) [97]. The obtained CoPx@NCNTs showed a high sensitivity of 802 μA μM^−1^ cm^−2^, a low limit of detection of 0.79 nM, and a broad linear range from 0.0025 μM to 1 μM towards p-nitrophenol (p-NP) with better stability and reproducibility in tap water and pond water. The developed structure showed better selectivity towards p-NP in the presence of various interfering ions due to the strong binding affinity of the hairy CNT structure with the benzene ring through strong π−π interactions, specifically recognizing the Co_x_P NPs and the unique pore structure-derived material.

A phosphorus-rich cobalt phosphide-embedded nitrogen-doped porous carbon (CoP_2_-N-C) was developed using ZIF-67 as a template through a pyrolysis and phosphidation process. The P-rich CoP_2_-N-C composite displayed better electrocatalytic properties towards chloramphenicol than the metal-rich counterparts, namely CoP and Co_2_P, due to the higher electronegativity in the presence of more P atoms, the high adsorption capacity, and the electrochemical reduction, thereby trapping the chloramphenicol [98,99,100,101]. The established CoP_2_-N-C-based sensor showed an increased peak current during the differential pulse voltammetry in PBS buffer with a low limit of detection of 0.044 μM.

Zhang et al. reported the synthesis of a nickel phosphide–graphene (Ni_2_P/G) composite by phosphorizing a Ni-MOF/graphene composite and using it as an electrocatalyst for the non-enzymatic glucose sensor (Figure 8b) [92]. The exposed nickel atom/specific sites with the framework in the Ni-MOF precursor easily transformed into Ni_2_P NPs, which were evenly distributed and immobilized into the conducting graphene sheets. This composite exhibited a low limit of detection of 0.44 μM and a wide linear range from 5 μM to 1.4 mM.

## 4. Applications: Electrochemical Sensing of Biomolecules

MOFs have a high surface area; tunable porosity; can be engineered to include active sites; can use the alteration of metal nodes and organic linkers to tailor their chemical and physical properties; and can be doped with other elements that tune the electrical and electronic properties, making them an excellent material for electrochemical applications, adsorption, and catalysis.

MOFs can be engineered to selectively adsorb specific analytes due to their customizable pore sizes and functional groups. This selective adsorption is essential for detecting particular molecules without the interference from other substances. MOFs can also replicate the catalytic functions of enzymes, enabling redox reactions of target analytes without requiring biological catalysts. This enzymeless method simplifies the process and avoids the potential instability linked with natural enzymes. The extensive surface area of MOFs offers numerous active sites for the adsorption and electrochemical reaction of analytes, thereby enhancing the sensor’s sensitivity and signal strength. Additionally, MOFs can be functionalized with various groups to increase their affinity for specific analytes or to boost their electrochemical properties, thus allowing for the precise detection of a wide range of analytes.

In this section, we will discuss the application of MOFs in glucose, dopamine, H_2_O_2_, ascorbic acid, and uric acid sensing.

### 4.1. Glucose Sensing

MOFs with various architectures, composites, metal centers, and functional organic ligands have been reported for efficient, selective, and sensitive glucose detection. Zhang et al. employed a hydrothermal approach to synthesize pyridine-modulated lamellar Ni-MOFs with ultrathin, well-defined 2D morphologies. Compared to bulk materials, the 2D lamellar Ni-MOF demonstrated enhanced electrochemical glucose sensing due to its larger surface area and higher active site density. This material exhibited a fast amperometric response, high sensitivity (907.54 µA mM^−1^ cm^−2^), and a wide linear detection range (0.5 to 2665.5 µM) for glucose [102].

Enzyme-based glucose detection has been widely reported to date with greater selectivity and sensitivity [47,103]. As an example, ZIF-based MOF-derived porous carbon was used as an enzyme immobilization platform for the fast detection of glucose [103,104]. However, enzyme immobilization technology faces challenges such as high costs, complex immobilization processes, and enzyme instability due to temperature and pH variations. To address these issues, recent research has demonstrated that MOF nanomaterials exhibit superior peroxidase-mimicking behavior, enhancing glucose detection selectivity and sensitivity [105]. Various MOF-derived metal oxides with different morphologies, including Co_3_O_4_, CuO, and NiCo_2_O_4_, have been developed for direct glucose sensing [51,57,106,107,108]. For instance, Muthurasu et al. reported MOF-derived Co_3_O_4_ grown on CuO nanowires supported by 3D porous copper foam (CuO NWs@Co_3_O_4_ electrode). The MOF was synthesized via in situ solution deposition followed by calcination, achieving excellent glucose oxidation in the linear range of 0.5 μM to 0.1 mM with a detection limit of 0.23 μM [52].

**Figure 9:**
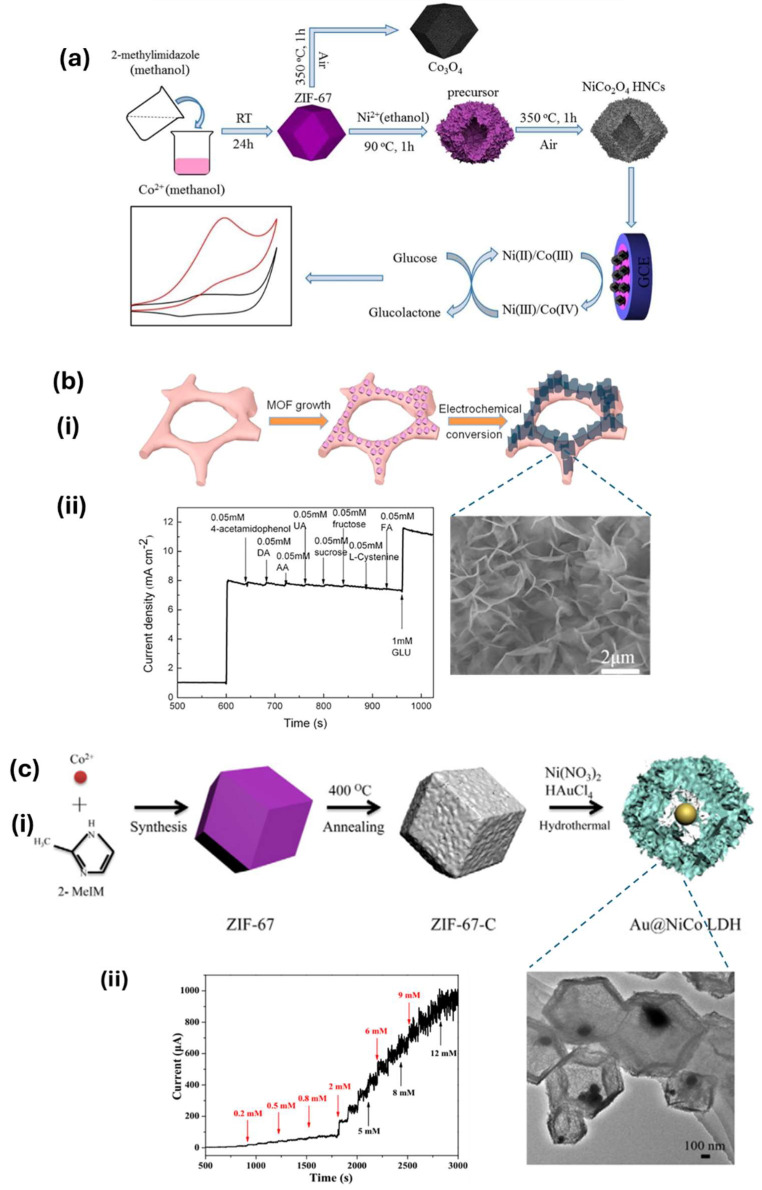
(**a**) Formation of NiCo_2_O_4_ hollow nanocages (HNCs) and Co_3_O_4_, as well as the catalytic mechanism for glucose oxidation [57]. (**b**) (i) Illustration of vertically oriented CuO nanosheets over Cu foam. (ii) Interference analysis conducted on the CuO/Cu electrode by introducing acetaminophen, ascorbic acid (AA), dopamine (DA), uric acid (UA), sucrose, fructose, L-cysteine, and folic acid into 0.1 M NaOH at 0.54 V [106]. (**c**) (i) Diagram of the synthesis of rattle-type Au@NiCo LDH hollow core–shell nanostructures. (ii) Amperometric response curves of Au@NiCo LDH/GCE upon the incremental addition of glucose concentrations ranging from 0.005 to 12 mM in 1.0 M NaOH at a potential of 0.5 V [109].

Cobalt-based ZIF-67 MOF-derived NiCo_2_O_4_ hollow nanocages (NiCo_2_O_4_ HNCs) were developed through the hydrothermal method followed by pyrolysis at 350 °C and were deposited on GCE for the electrochemical oxidation of glucose in alkaline media. The developed electrode exhibited a fast response time of 1 s, and good interference resistance with a sensitivity and detection limit of 1306 μA mM^−1^ cm^−2^ and 27 nM, respectively (Figure 9a) [57]. Zhang et al. designed a vertically aligned CuO nanosheet film on conducting Cu foam by electrochemically growing Cu MOFs followed by electrochemical conversion, as shown in Figure 9b(i). The vertically aligned architecture exhibited a sensitivity of 33.95 mA cm^−2^ mM^−1^ with a detection limit of 330 nM, and showed a good anti-interference ability towards various analytes, including acetaminophen, DA, AA, UA, sucrose, fructose, L-cysteine, and folic acid, as shown in Figure 9b(ii).

**Figure 10:**
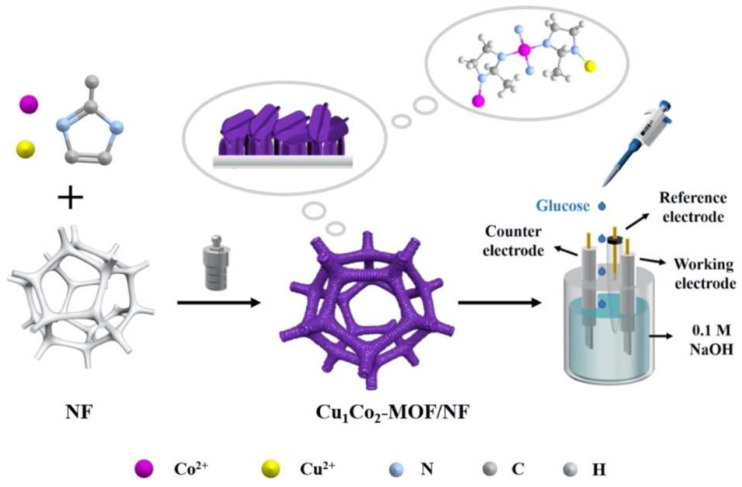
Fabrication of Cu1Co2-MOF/NF and glucose detection on the electrode [111].

Bimetallic metal oxides derived from MOFs have also been incorporated for the non-enzymatic electrochemical detection of glucose, owing to the synergistic effect of the metal elements, and their improved electrical conductivity compared to single metal oxides [110]. Du et al. synthesized bimetallic MOFs in situ on nickel foam (NF) using an inexpensive one-pot hydrothermal technique. Electrochemical measurements revealed that the Cu1Co2-MOF/NF composite electrode displayed a high sensitivity of 8304.4 μA mM^−1^ cm^−2^ and an LOD of 0.023 mM (S/N = 3) [111]. Further, it demonstrated an excellent anti-interference performance toward ascorbic acid (AA), uric acid (UA), dopamine hydrochloride (DA), and sodium chloride (NaCl). These outcomes suggest that Cu1Co2-MOF/NF is a viable material for high-performance glucose sensing, as shown in Figure 10 [111].

Apart from bimetallic compounds, MOF-derived LDHs have been widely used as electrode materials for glucose sensing due to their versatility in chemical composition, tunable structures, unique anion exchange, and intercalation properties [59,112]. In this context, cobalt-based ZIF-67 was used as a sacrificial template to synthesize Co_x_Ni_1−x_-LDHs’ yolk-shell structure. Moreover, by tuning the composition of cobalt and nickel, the yolk-shell structure was converted into a hollow structure, which showed a better electron transfer pathway and a relatively higher responsive current leading to a low detection limit (3.1 µM) and high sensitivity (242.9 μA mM^−1^ cm^−2^) [113].

MOF-derived carbon–metal oxide nanocomposites have been fabricated for the fast response and sensitive electrochemical detection of glucose [106]. In this context, a silver NP-decorated nickel-based MOF-74 nanocomposite was developed and deposited on a GCE to detect glucose in a concentration range between 0.01 and 4 mM with a detection limit of 4.7 μM [114]. Additionally, Yuniasari et al. developed a graphene-modified Co-MOF for glucose detection using an electrochemical method. The Co-MOF’s large surface area and porous structure, combined with graphene’s conductivity, enhanced the sensor’s current response. The Co-BDC-3Gr composite demonstrated a sensitivity of 100.49 μA mM^−1^ cm^−2^, with an LOD of 5.39 μM and excellent stability [115].

A NiCo LDH nanosheet/graphene nanoribbon (GNR) nanocomposite was developed by mixing GNR and NiCo LDH nanosheets. The NiCo nanosheets were synthesized on the surface of sacrificial ZIF-67 MOF using a nickel precursor and the sol–gel reaction. The synthesized nanocomposite-modified GCE possessed enhanced mechanical stability and electrical conductivity [112]. Chu et al. used NiCo-LDH to fabricate a hollow NiFe_2_O_4_-NiCo-LDH@rGO cube using the hydrothermal method and pyrolysis process. The material was synthesized by using the Ni-Fe bimetallic organic framework (NiFe-MOF) to prepare hollow NiFe_2_O_4_ nanocubes, followed by the growth of NiCo LDH nanowires on the surface of the NiFe_2_O_4_ nanocubes with the attachment of reduced graphene oxide through the hydrothermal method. The nanocomposite cubes exhibited glucose detection in a wide linear range of 3.500 × 10^−5^ − 4.525 × 10^−3^ M, with a detection limit of 12.94 × 10^−6^ M [116].

Noble metal-layered double hydroxide (LDH) composites derived from MOFs enhance glucose sensing by combining their high conductivity and catalytic activity. These composites offer increased surface area, improved electrochemical properties, and sensitivity, enabling accurate, real-time glucose detection with low detection limits. Wang et al., as an example, developed a Au@NiCo LDH hollow core–shell nanostructure using the etching of ZIF-67 MOFs as a sacrificial template, followed by the immobilization of Au NPs inside the cavities of the hollow architecture, as shown in Figure 9c(i) [109]. The Au@NiCo LDH-modified GCE showed the electrochemical detection of glucose in a linear range of 0.005–12 mM (Figure 9c(ii)) with a sensitivity and a detection limit of 864.7 μA mM^−1^ cm^−2^ and 0.028 μM, respectively. Similarly, the CuCo-BTC/Au/MWCNTs composite was synthesized on copper foam (CuCo-BTC/Au/MWCNTs/CF) using a simple hydrothermal method to fabricate a CuCo-BTC sensor for glucose sensing [117]. The composite’s improved electrochemical performance was attributed to the synergistic effects of the bimetallic active sites, MWCNT’s higher conductivity and surface area, and gold’s strong catalytic activity. These combined properties resulted in more active sites, faster charge transfer, and higher electrocatalytic activity. The developed sensor possessed a linear detection range of 0.01–5 mM and 5–9 mM, a sensitivity of 1.029 mA mM^−1^ cm^−2^, and a detection limit of 3.4 μM (S/N = 3) for glucose. Furthermore, the sensor revealed good selectivity, repeatability, and stability.

### 4.2. Dopamine Sensing

MOFs are an exceptional candidate for the ultralow concentration detection of dopamine through electrochemical means. The modification of electrodes using various types of MOFs leads to a decrease in the electrode potential of the analytes as well as improving redox kinetics, which enhances the electrochemical sensing of DA. Copper-based MOFs have been widely used to modify the GCE for the sensitive and selective electrochemical detection of DA with greater electrochemical activity [118,119]

A two-dimensional conductive MOF was designed using the redox-active organic linker 2,3,6,7,10,11-hexahydroxytriphenylene with copper metal centers to fabricate an electrochemical sensor to detect DA in the concentration range of 5.0 × 10^−8^ to 2.0 × 10^−4^ M with a detection limit of 5.1 nM. The low detection limit of DA was mainly attributed to the π-conjugated MOF with high electrical conductivity, better charge storage capacity, and also the redox-active linker with high conjugation, which enabled the charge transfer between the metal nodes and organic linkers [120].

The functionalization of MOFs has also been incorporated to increase sensitivity. Recently, an electron-deficient bipyridinium moiety which forms donor–acceptor charge-transfer complexes with electron-rich species such as dopamine has been used to develop Zr-based MOFs, such as UiO-67-MQ and UiO-67-DQ, where MQ = N,N′-dimethyl-2,2′-bipyridinium, and DQ = N,N′-ethylene 2,2′-bipyridinium, for enhanced electrochemical sensitivity towards DA. The strong electrode–analyte interaction results in better sensing performance compared to non-functionalized MOFs [121].

Apart from pristine and functionalized MOFs, a nanocomposite of carbon–MOFs or NP–MOFs has been used to improve the conductivity and catalytic and mechanical properties for the enhanced electrochemical detection of DA with greater sensitivity and faster response times [122,123]. Moreover, the incorporation of MWCNTs into functionalized MOFs can further improve the sensitivity and decrease the detection limit by an order of magnitude, due to the better electron-transfer kinetics [121,124].

Recently, a nanocomposite of electro-reduced graphene oxide (ERGO) with copper-based MOF HKUST-1 was formed through electrochemical deposition, which showed excellent electrochemical activity towards DA due to the enhanced surface area, the high electrical conductivity of ERGO, and the optimum porosity. The developed electrochemical sensor exhibited a low limit of detection of 0.013 μM and a wide linear sensing range of 0.2 μM to 300 μM [125]. Further, a copper terephthalate MOF-GO nanocomposite material was developed through π−π stacking, Cu−O coordination, and hydrogen bonding, which showed the synergistic effect of the electron mediation of copper terephthalate and reduced graphene [126].

**Figure 11:**
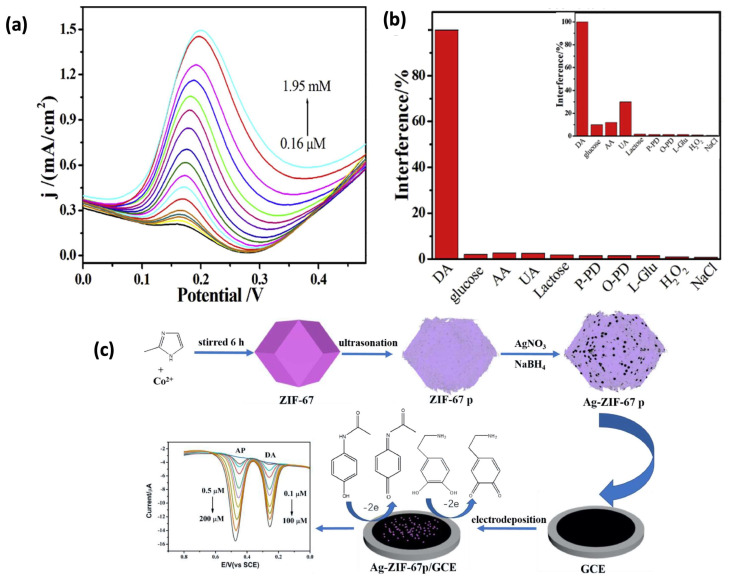
(**a**) Differential pulse voltammetry (DPV) response of the MIPs/CuCo_2_O_4_@carbon/3D−KSC integrated electrode in 0.1 M PBS (pH = 7.0) with various DA concentrations [127]. (**b**) Histogram showing the current response proportions of the MIPs/CuCo_2_O_4_@carbon/3D-KSC electrode and CuCo_2_O_4_@carbon/3D−KSC electrode in 0.1 M PBS (pH = 7.0) with 0.5 mM DA and potential interfering substances [127]. (**c**) Fabrication of Ag-ZIF-67 nanopinna-modified GCE and its application in the detection of AP and DA [129].

A hybrid CuCo_2_O_4_@carbon nanocomposite on 3D porous carbon was derived by Wang et al. using a Cu-Co-ZIF precursor and a molecular imprinted electrochemical sensor was developed, which exhibited a sensitivity of 720.8 mA mM^−1^ cm^−2^ with a detection limit of 0.16 mM [127]. The DPV curves showed an increase in the current response of the MIPs/CuCo_2_O_4_@carbon/3D-KSC integrated electrode as the concentration of DA increased (Figure 11a). The chitosan film was electrodeposited on CuCo_2_O_4_@carbon/3D-KSC and MIPs were formed. Moreover, the fabricated electrode showed good anti-interference properties, as illustrated in Figure 11b [127].

Noble metal MOF composites enhance dopamine sensing by improving conductivity, catalytic activity, and electron transfer [46]. A nanocomposite comprising ZIF-90 MOF and the Pt_41_Rh_59_ alloy nanocatalyst was designed for the electrochemical detection of DA with a low detection limit of 1 nM. The developed MOF possessed a free aldehyde group in the organic ligand which could covalently bind and adsorb the dopamine [128]. Similarly, silver NP-decorated ZIF-67 composite was developed by Tang et al. using the in situ reduction of AgNO_3_ precursor on the surface of MOF, as shown in Figure 11c. The developed nanocomposite had a large number of active sites, high conductivity, and better catalytic properties to enhance the electrochemical performance of the dopamine sensor with a detection limit of 0.05 μM [129]. Moreover, a mixed noble metal in MOF was used by Hira et al. to develop a highly sensitive method for DA detection via a AgPd@Zr-MOF composite. The exceptional DA sensing performance was attributed to its large specific surface area and the abundance of electroactive sites. The fabricated sensor exhibited a detection limit of 0.1 μM, a sensitivity of 10.26 μA μM^−1^ cm^−2^, and a linear DA concentration range of 2–42 μM [130].

Similarly, Co/Co-N@NPCs were fabricated using the pyrolysis of ZIF-67 MOF under an inert atmosphere and were modified with CPE to detect DA in two linear ranges from 10 nM to 50 μM, and 50 μM to 500 uM, with high selectivity over various types of biomolecules and a low detection limit of 6 nM. Moreover, the presence of the Co^2+^ ion in ZIF-67 MOF could improve the graphitization of carbon, which enhanced the electrochemical kinetics and promoted the electron transfer and fast electrochemical response [131].

**Figure 12:**
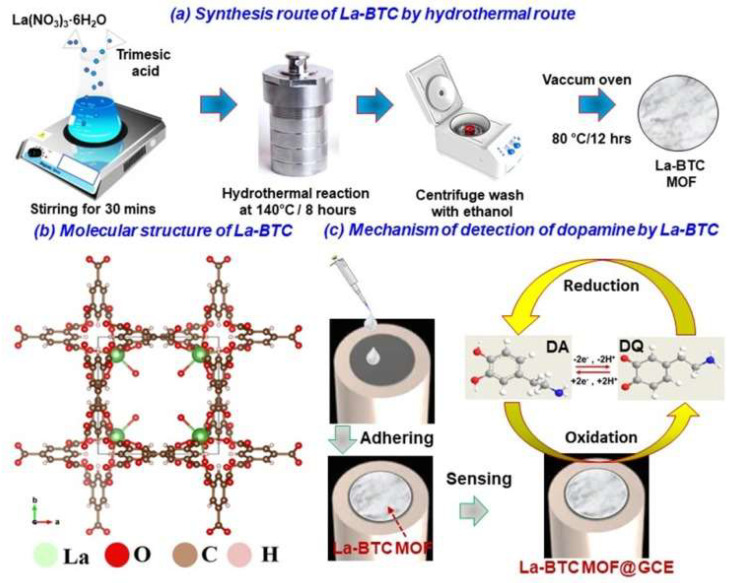
(**a**) Synthesis of La-BTC/CNT by the hydrothermal route; (**b**) the molecular structure of La-BTC; and (**c**) the schematic route of the La-BTC/CNT-modified electrode for sensing dopamine [132].

Rajaitha et al. demonstrated the direct integration of CNT and lanthanum MOF (La-BTC) hybrids onto an electrode for the electrochemical sensing of dopamine. The hydrothermal technique was used to prepare the La-BTC/CNT composite, as shown in Figure 12a–c. CV and LSV were used to examine the electrochemical behavior of dopamine detection. The La-BTC/CNT composites showed better electrocatalytic activity than La-BTC alone. It was found that the limits of quantitation (LOQ) and LOD were 0.24 µM and 0.073 µM, respectively. The modified electrode sensitivity was measured at 2.953 μA μM^−1^cm^−2^. Furthermore, the La-BTC/CNT electrode exhibited strong stability, indicating that these nanohybrids were viable options for dopamine detection that did not use enzymes [132].

### 4.3. H_2_O_2_ Sensing

H_2_O_2_ is an important compound that can play a vital role in cell damage, the human immune system, enzymatic reactions, cellular *signaling* transduction, environmental contamination, and damage if in excess, and there are risks of oxidative degradation to certain sensitive pharmaceuticals [133,134,135]. MOFs offer a large number of active sites to boost catalytic activity and play an important role in the enzymatic as well as the non-enzymatic detection of H_2_O_2_ [136,137,138,139,140].

**Figure 13:**
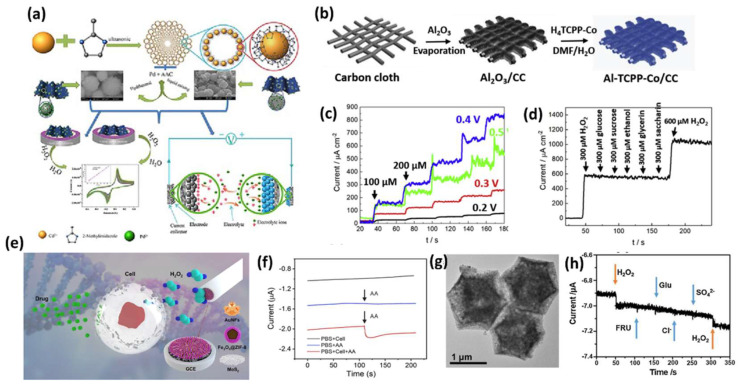
(**a**) Diagrammatic illustration of the Pd nanocubes@CdIF−8 catalyst fabrication approach for the extremely effective electrocatalytic detection of H_2_O_2_ [144]. (**b**) Illustration of the surface functionalization of carbon cloth with Al−CPP−Co MOF [146]. (**c**) Amperometric curves of Al−TCPP−Co/CC recorded at various working potentials [146]. (**d**) Current response over time for Al−TCPP−Co/CC during interference analysis in the presence of ethanol, glycerin, glucose, sucrose, and saccharin [146]. (**e**) Schematic diagram of Au nanoflowers (NFs) and Fe_3_O_4_@ZIF−8-MoS_2_ nanocomposite-modified GCE for the electrochemical detection of H_2_O_2_ derived from living cells under pharmacological stimulation [148]. (**f**) Amperometric responses of Au NFs/Fe_3_O_4_@ZIF−8-MoS_2_/GCE to the addition of 400 μM ascorbic acid (AA), both with and without H9C2 cells, as well as in the presence of H9C2 cells without AA (cell count: 2 × 10^6^) [148]. (**g**) Transmission electron microscopy (TEM) images of Co_3_O_4_/NCNTs [149]. (**h**) Amperometric response of the Co_3_O_4_/NCNT electrode at −0.2 V following the sequential introduction of 0.2 mmol L^−1^ hydrogen peroxide (H_2_O_2_), fructose (FRU), glucose (Glu), chloride (Cl^−^), and sulfate (SO_4_^2−^) into 0.1 mol L^−1^ phosphate-buffered saline (PBS) at pH 7.0 [149].

Various types of MOF–carbon composites have been developed for H_2_O_2_ sensing such as ZIF-67/rGO, ZIF-8/rGO, and Fe-MOF/rGO [123,141,142,143]. Additionally, various noble metal–MOF composites have been explored for H_2_O_2_ sensing. Recently, Li et al. synthesized a Pd nanoparticle-decorated Cd(2-methylimidazole)_2_ MOF, anchored on functionalized activated carbon through hydrogen bonding and π-π interactions, forming Pd@CdIF-8@AAC, as shown in Figure 13a [144]. This composite exhibited a synergistic effect, enhancing adsorption and catalytic activity toward H_2_O_2_. Chen et al. synthesized a self-supporting electrochemical sensor by electrodepositing metal nanoparticles onto Zn-MOFs. AgNPs exhibited superior electrocatalytic activity for H_2_O_2_ reduction, especially with 2D Zn-MOFs, which enhanced their conductivity and stability. The Ag/2D Zn-MOF electrode achieved a broad detection range (5.0 μM–70 mM) and a 1.67 μM LOD [145].

Gao et al. prepared a carbon–MOF composite using redox-active cobalt porphyrin-based MOF anchored on a functionalized carbon cloth substrate, as shown in Figure 13b, which shows the electrochemical detection of H_2_O_2_ in the range of 1–1000 mM, with a detection limit of 0.7 mM [146]. The amperometric responses at various potentials are improved with increasing applied voltages, as shown in Figure 13c; moreover, there are no significant fluctuations in the current signals with the subsequent addition of the interfering agents, as shown in Figure 13d.

The combination of MOFs with magnetic NPs such as Fe_3_O_4_ was used to construct magnetic MOF nanocomposite nanozymes, which have the advantage of the high peroxidase-like activity of Fe_3_O_4_ NPs and the easy separation of the MMOF from the reaction solution [138,142,147]. In this context, an electrochemical sensor was prepared by Lu et al. in the living cells using a GCE-modified Au NFs/Fe_3_O_4_@ZIF-8-MoS_2_ nanocomposite, as shown in Figure 13e. The gold NFs were electrodeposited on Fe_3_O_4_@ZIF-8 MOF which was supported on MoS_2_ nanosheets to maintain the surface active sites, the electrocatalytic activity, and to enhance the detection signals by 250 times compared to bare GCE, and showed a detection limit of 0.9 μM [148]. Figure 13f shows the change in electrochemical signal with the addition of 400 μM AA into PBS solution containing H9C2 cardiac cells.

A hollow framework of cobalt oxide nitrogen-doped CNTs, namely Co_3_O_4_/NCNTs, was synthesized through the pyrolysis of ZIF-67 MOF in a H_2_/Ar atmosphere followed by calcination in the air, where the MOF acted as a self-sacrificial template which exhibited the non-enzymatic electrochemical detection of H_2_O_2_, with a detection limit of 1mmol L^−1^ and a sensitivity of 87.40 mA mmol L^−1^ cm^−2^ (Figure 13g) [149]. Also, as a sensor electrode, the Co_3_O_4_/NCNTs showed good anti-interference ability towards glucose, fructose, chlorine ions, and SO_4_^2−^ ions, as shown in Figure 13h [149].

MXene-MOF composites enhance H_2_O_2_ sensing by combining MXene’s high conductivity with MOF’s porous structure and catalytic activity. This synergy improves electron transfer, sensitivity, and selectivity, enabling efficient electrochemical detection. In this regard, Cheng et al. synthesized a Cu-MOF-MXene composite to develop a sensitive electrochemical sensor on GCE for detecting H_2_O_2_. Because of the excellent electrical conductivity of MXene, the catalytic properties of Cu-based nanomaterials, and the structural characteristics of MOFs, the Cu-MOF-MXene-GCE sensor revealed remarkable electrocatalytic activity during the reduction of H_2_O_2_. The Cu-MOF/MXene/GCE sensor demonstrated a broad linear detection range from 1 μmol L^−1^ to 6.12 mmol L^−1^ with an LOD of 0.35 μmol/L [150]. Arjun et al. functionalized carbon cloth (CC) with a novel composite of Nb_2_CT_x_ MXene and Prussian Blue (PB) for the electrochemical detection of H_2_O_2_. The developed sensor demonstrated excellent selectivity, effectively detecting H_2_O_2_ even in the presence of interfering species such as dopamine, ascorbic acid, uric acid, and sodium chloride. Additionally, the sensor exhibited remarkable storage stability, retaining 97% of its activity after one week of storage [151].

### 4.4. Ascorbic Acid (AA) Sensing

MOFs offer high porosity, large surface area, and tunable active sites, making them ideal for ascorbic acid (AA) sensing. Various MOFs and their composites, including Ni-MOFs, Cu-MOFs, Zn-MOFs, and Ce-MOF have been explored for this purpose [152,153]. Conductive MOFs, MOF–carbon hybrids, and noble metal–MOF composites enhance electrocatalytic activity, electron transfer, and stability. Their robust stability and catalytic efficiency make them promising for real-time AA detection applications [154].

Recently, Shen et al. synthesized HKUST-1 nanosheets using a solvent-based method at an ambient temperature and immobilized them on an ITO electrode for ascorbic acid sensing. The sensor exhibited a diffusion-controlled, pH-dependent mechanism with a linear response from 0.01 to 265 mM and a 3 mM LOD, attributed to its porous structure enhancing electron transfer [155]. Conductive Ni_3_(2,3,6,7,10,11-hexaiminotriphenylene)_2_ (Ni_3_(HITP)_2_) nanorods were synthesized and used to fabricate an enzyme-free electrochemical sensor for ascorbic acid (AA) detection. The sensor, integrated with a smartphone app, exhibited a sensitivity of 0.814 μA μM^−1^ cm^−2^ in the linear range between 2 and 200 μM with a detection limit of 1 μM, enabling real-time vitamin C monitoring in sweat [153].

Apart from pure MOFs, MOF–carbon composites have also been reported due to the synergy between MOFs and carbon materials, which enhances electron transfer, catalytic activity, and sensitivity [156]. These composites offer strong adsorption, rapid responses, high surface areas, excellent conductivity, and improved stability, making them ideal for the accurate and reliable electrochemical detection of ascorbic acid [157,158]. Li et al. developed a ZIF-65@CNT-nanohybrid-composite-modified GCE sensor in PBS buffer using the DPV method. The porous ZIF-65 structure, nitro group oxidation sites, and CNT conductivity enhanced redox mediation, enabling efficient AA reduction with a detection limit of 1.03 mM [35].

Bimetallic metal oxide–carbon composites derived from MOF were also used for AA sensing by combining the high catalytic activity of bimetallic MOFs with the excellent conductivity of carbon materials. This synergy improved electron transfer, broadened the reaction scope, provided strong stability, and increased sensitivity and selectivity. In this context, a bimetallic MOF carbon composite, such as Co_3_O_4_/Fe_3_O_4_/C-loaded g-C_3_N_4_, was derived from CoFe-based bimetallic MOFs through high-temperature carbonization followed by mixing with nitrogen-containing melamine and heat treatment, where the organic ligands were converted into mesoporous carbon while the metal oxides were initiated from the oxidation of metal coordination centers. The developed nanocomposite was modified with GCE, which exhibited an LOD of 12.55 μM through the DPV method [159].

Noble metal-doped carbon–MOF composites enhance AA sensing by improving electrical conductivity, increasing active sites, and promoting faster electron transfer. Doping metals like gold, platinum, or silver into carbon–MOF structures boosts catalytic activity, conductivity, and stability, leading to better sensitivity, selectivity, and durability for AA detection. For example, Krishnan et al. developed an electrode on indium tin oxide using the g-C_3_N_4_/NC@GC/h-ATS nanocomposite sensing material through the drop-casting method for the simultaneous electrochemical detection of AA, DA, and UA. NC@GC was derived from the carbonization of the core–shell ZIF-8@ZIF-67 MOF structure, whereas the h-ATS nanostructure containing Ag@TiO_2_/SnO_2_ was derived from the sacrificial hierarchical mesoporous silica (SBA-15) template through the calcination process. The developed electrode-containing nanocomposite material exhibited well separated and elevated oxidation peaks towards AA, DA, and UA, with an LOD of 0.02, 0.01, and 0.06 μM for AA, DA, and UA, respectively [160].

A flexible electrochemical sensor electrode poly(o-PD)/ZIF-67/CC was developed using ZIF-67 MOF-decorated conducting carbon cloth with the electro-polymerization of o-phenylenediamine for a high sensitivity of 959.9 μA mM^−1^ cm^−2^ with a detection limit of 0.019 μM. Compared to the developed sensor electrodes with GCE, the flexible electrodes showed the negligible detachment of sensing materials, leading to consistent sensing performance [161].

**Figure 14:**
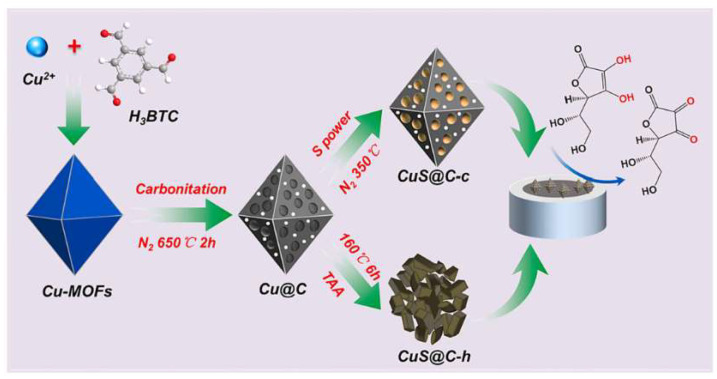
MOF-derived 3D porous octahedral structure of copper sulfide (CuS)/carbon composites (CuS@C-c) for electrochemical detection of ascorbic acid [162].

CuS/carbon composites (CuS@C-c) with a porous octahedral structure were synthesized by Dong et al. through pre-carbonization followed by the sulfidation of Cu-MOF precursors. This method encapsulated CuS in the carbon matrix in situ, resulting in good conductivity and strong structural integrity. CuS@C-c exhibited superior electrochemical performance compared to hydrothermally produced CuS@C-h with disordered structures. CuS@C-c-modified electrodes effectively oxidized AA over a wide linear range (0.1–1000.0 μmol/L), with an LOD of 0.03 μmol/L [162]. The improved performance was attributed to the synergistic effects of CuS and the carbon matrix, along with their 3D open porous structure, offering numerous accessible active sites and enabling rapid electron transport (Figure 14).

### 4.5. Uric Acid Detection

The presence of uric acid (UA) in excess amounts creates several diseases, including arthritis, gout, hyperuricemia, etc. The recommended concentration of UA in human urine lies between 1.4 mM and 4.46 mM. To detect such lower concentrations with greater accuracy and a low detection limit, various types of MOFs, their derivatives, and MOF–nanocomposites have been employed as outstanding electrode materials that can perform fast detection with higher sensitivity [163].

ZIF MOF-derived porous carbon with tunable pores, a high surface area, and graphitization enhanced electrochemical sensitivity. Hybrid materials with heteroatom-doped MOFs or MOF-derived metal–carbon composites improved UA detection. Nitrogen- and cobalt-doped carbon nanoparticles from ZIF-67 on GCE detected DA and UA with sensitivities of 1130.3 and 610.7 μA mM^−1^, respectively [160,164,165,166].

**Figure 15:**
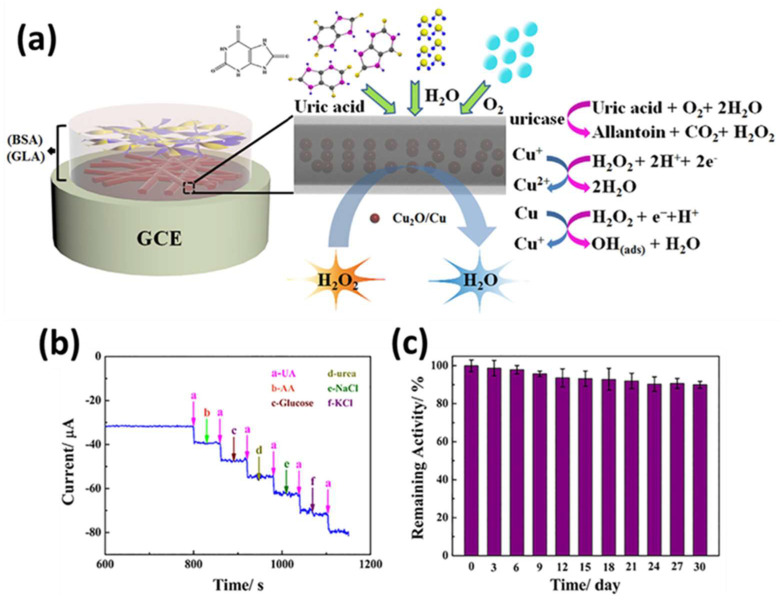
(**a**) Detection of uric acid (UA) using Cu_2_O/Cu@C core–shell nanowires on GCE electrode. (**b**) Determination of selectivity of developed UA biosensors. (**c**) Assessment of stability of fabricated UA biosensors after one month of storage [167].

A copper-based MOF HKUST-1 was used as a template to fabricate porous Cu_2_O/Cu@C core–shell nanowires through thermal decomposition, which exhibited the synergistic advantages of Cu, C, and Cu_2_O, including the excellent catalytic performance of cuprous oxide, the high conductivity of carbon, and the porous architecture of the material, resulting in better electron transfer, improved sensitivity, and anti-interference ability (Figure 15a). Moreover, the controlled carbonization process endowed the optimal wrapping of Cu and CuO within the porous nanowire shell, and lowering the electrochemical working potential caused the better selectivity of UA over other biomolecules, as shown in Figure 15b. The sensitivity, linear range, and detection limit of the fabricated sensors were 330.5 μA mM^−1^ cm^−2^, 0.05 to 1.15 mM, and 0.4 μM, respectively [167]. Moreover, the fabricated sensor displayed good stability after 1 month, as shown in Figure 15c.

Bimetallic MOFs enhance uric acid detection by providing a high surface area, tunable porosity, and multiple catalytic sites. Incorporating metals like Ce into Zn-MOFs improves charge conduction, sensitivity, and stability. These composites, used on GCE sensors, achieved low detection limits and wide linear ranges, making them effective for electrochemical sensing [159]. In this respect, a GCE-modified porous carbon electrode was developed via the carbonization of bimetallic ZIFs (ZIF-8 and ZIF-67) followed by etching, achieving a detection limit of 0.83 μM (linear range: 2.0–110 μM) for real serum analysis using DPV [168]. Additionally, a Ce@Zn-MOF/GCE sensor synthesized via the solvothermal incorporation of Ce salts exhibited a 0.51 ng LOD and a 0–300 ng/mL range for UA detection, enhanced by Ce-induced charge conduction and structural stability [169].

Enzymatic uric acid detection using MOFs enhances sensitivity and selectivity by immobilizing uricase within MOFs. MOFs provide a stable, high-surface-area matrix for enzyme activity, improving electron transfer and detection accuracy. These biosensors are used in medical diagnostics, clinical monitoring, and point-of-care testing for hyperuricemia and gout [170]. A porous cerium oxide–carbon nanocomposite, derived from a Ce-based MOF and immobilized with uricase via physical adsorption, was used to construct a uric acid biosensor on GCE. This sensor exhibited a sensitivity of 220.0 μA cm^−2^ mM^−1^ at −0.4 V vs. SCE [170]. Additionally, Mashkoori et al. developed an electrochemical biosensor by modifying CPE with histidine-tagged urate oxidase (H-UOX) and Ni-MOF, achieving a detection range of 0.3–140 µM and a 0.084 µM LOD. The biosensor demonstrated a fast response, and high efficiency, repeatability, and reproducibility [171].

Noble metal MOF composites enhance uric acid detection by offering high conductivity, catalytic activity, and stability. Incorporating metals like Au, Ag, or Pt into MOFs improves electron transfer, sensitivity, and selectivity, making them ideal for electrochemical biosensors with low detection limits and wide linear ranges. In this context, Zhou et al. fabricated a Au@Cu-MOF sensor with enhanced electrocatalytic activity for the electrochemical oxidation of dopamine (DA) and uric acid (UA). The sensor exhibited a linear detection range of 10–1000 μM, with sensitivities of 0.231 μA μM^−1^ cm^−2^ for DA and 0.275 μA μM^−1^ cm^−2^ for UA. The detection limits were 3.40 μM for DA and 10.36 μM for UA [172].

## 5. Conclusions and Perspectives

In summary, we presented a comprehensive review of the development of various types of MOF-based materials, including pure MOFs; MOF-derived metal oxides, metal sulfides, metal phosphides, and metal nitrides; MOF–carbon composites; MOF–noble metal composites; and MOF–polymer composites. We also discussed their applications in various biosensors for detecting glucose, dopamine, H_2_O_2_, ascorbic acid, and uric acid.

These advanced materials exhibit unique structural properties, high surface areas, and excellent conductivity, which enhance their electrochemical performance. The inclusion of carbon enhances electrical conductivity, reduces the background current, and optimizes conductive pathways. Noble metals, when immobilized on or within MOFs, prevent agglomeration, improve stability, and enhance catalytic and sensing capabilities due to their larger surface area and uniform dispersion. Metal oxide properties can be finely tuned through controlled calcination, creating more active sites and improving electrochemical performance. LDHs, with their biocompatibility and increased active sites, present another promising material for biosensor applications. The high surface area and adsorption capabilities of MOFs make them an ideal backbone for conducting polymers, facilitating rapid electron/ion diffusion. MOF-derived metal sulfides, phosphides, and nitrides offer superior electrical conductivity and structural stability, making them excellent candidates for electrochemical sensing. TMSs, with their 2D layered structure and appropriate bandgap, provide large active sites and better electrical conductivities, improving charge transport and electrochemical sensitivity. Functionalizing MOFs further enhances their sensitivity by improving electrode–analyte interactions. For instance, sulfur-functionalized MOFs show improved sensing performance due to electrostatic interactions, hydrogen bonding, and enhanced dopamine oxidation. These advancements in material engineering underscore the significant potential of MOF-based composites in creating highly sensitive, stable, and efficient electrochemical sensors for detecting glucose, dopamine, H_2_O_2_, ascorbic acid, and uric acid.

**Future Perspectives:**

In the near future, MOF materials and their composites with various carbons, metal oxides, sulfides, nitrides, and phosphides are poised to become pivotal in the electrochemical sensing of a diverse range of analytes, such as glucose, dopamine, H_2_O_2_, ascorbic acid, and uric acid. MOFs derived from binary and ternary metal nitrides, phosphides, and sulfides, despite their high electrical conductivity and surface area, are still in their early stages of development for biosensing applications. The unique properties of MOFs, such as their high surface area, tunable pore size, and versatile functionality, hold immense promise for the creation of electrochemical sensors that are highly sensitive and selective. However, there are still several obstacles that need to be overcome. A primary challenge is the stability and reproducibility of MOF-based sensors, particularly under adverse environmental conditions. In addition, the limited availability and high cost of certain MOF materials can impede their practical application. To surmount these challenges, there is a need for further research and development to create novel MOF materials with improved stability, reproducibility, and cost-effectiveness. Furthermore, innovative approaches for the synthesis and integration of MOFs with other materials must be explored to enhance the sensing capabilities of MOF-based sensors. Overall, the development of MOF-based electrochemical sensors holds tremendous potential for a wide range of applications in areas such as healthcare, environmental monitoring, and food safety. However, continued research is needed to overcome current challenges and to further optimize the performance of these sensors for practical use.

## Figures and Tables

**Table 1:** Quantitative comparison of detection limits and sensitivity in MOF-derived oxides and sulfides.

Materials	Biosensor Target	LOD	Sensitivity	Key Properties	References
CuO derived from Cu-MOF	Glucose	70 nM	NA	Detection Range: 500 nM to 5 mM	[51]
CuCo_2_O_4_ nanowire arrays	Glucose	0.5 µM	3.93 mA mM^−1^ cm^−2^	Detection Range: 500 nM to 5 mM, Binder-free Electrode	[83]
CuO_x_@Co_3_O_4_ core–shell nanowires	Glucose	36 nM	27,778 μA mM^−1^ cm^−2^	Core–Shell Structure, Hierarchical Porosity, Well-Defined Nanowires, High Conductivity	[53]
3D CuO NWs@Co_3_O_4_ nanostructures	Glucose	0.23 µM	6082 μA/μM	Binder-Free Architecture, 3D Porous Structure, Aligned CuO Nanowires	[52]
NiCo_2_O_4_ hollow nanocages	Glucose	27 nM	1306 μA mM^−1^ cm^−2^	High Electrical Conductivity, Hollow Nanocage Structure, Strong Structural Stability	[57]
ZnCo_2_O_4_ micro-rice-like microstructure	Glucose	5 µM	436.1 μA mM^−1^ cm^−2^	High Active Site Density, Enhanced Electrical Conductivity, Porous Micro-Rice Structure, Surface Area: 25 m^2^ g^−1^	[58]
CC@CCH MOF LDH	Glucose	110 nM	4310 μA·mM^−1^·cm^−2^	Three-Dimensional Hierarchical Structure, High Porosity	[59]
NiS nanocubes	H_2_O_2_	1.72 µM	NA	Surface Area: 139 m^2^g^−1^, Detection Range: 4–40 µM, Hollow and Porous Nanostructure	[78]
CoS_x_@CdS polyhedron	Hg(II)	2.0 pM	NA	Detection Range: 0.010–1000 nM, Turn On Photoelectrochemical Sensor	[79]
MWCNT with CoS nanoparticles	Glucose	5 μM	15 mA M ^−1^ cm^−2^	Detection Range: 8μM to 1.5 mM, High Electrical Conductivity	[84]
rGO/CuS composite	Glucose	1.75 nM	NA	Detection Range: 0.1–100 mM, High Electrical Conductivity	[85]
Hollow sphere-structured nickel sulfide (HS-NiS) nanomaterials	Lactic Acid	0.023 μM	0.655 μA μM^−1^ cm^−2^	Detection Range: 0.5 to 88.5 μM, Selectivity Against Uric Acid, Ascorbic Acid, Paracetamol	[86]
Nanoflake-like SnS_2_ matrix	Glucose	0.01 mM	7.6 mA M ^−1^ cm^−2^	Detection Range: 0.025 to 1.1 mM	[87]

## Data Availability

Not applicable.

## Glossary for MOF Acronyms

MOF	Metal–Organic Framework
MIL	Materials of Institute Lavoisier (e.g., MIL-101, MIL-53)
UiO	University of Oslo (e.g., UiO-66, UiO-67)
ZIF	Zeolitic Imidazolate Framework (e.g., ZIF-8, ZIF-67)
HKUST	Hong Kong University of Science and Technology (e.g., HKUST-1)
PCN	Porous Coordination Network (e.g., PCN-222, PCN-224)
IRMOF	Isoreticular Metal–Organic Framework (e.g., IRMOF-1, IRMOF-3)
DUT	Dresden University of Technology (e.g., DUT-49)
